# The American Medical Association—Atlantic City—Lehigh Valley R. R.

**Published:** 1904-05

**Authors:** 


### The American Medical Association—Atlantic City—Lehigh Valley R. R.

THE fifty-fifth annual session of the American Medical Association will convene at Atlantic City on Tuesday, June 7, 1904, to continue during four days. There are several reasons why the place selected should prove advantageous and attract a large attendance. One of the important features to be considered in selecting the place of meeting of this association is that of hotel accommodations. At Atlantic City this subject need give no anxiety either in reference to quantity or quality. There are plenty of them and there is no place in the world where guests can be made more comfortable either as to quarters, or quality of table. Moreover, there are first class boarding houses in abundance with lower rates than hotels, for those who prefer them.

The next item of importance is accessibility and here, again, there is no room for doubt. The railways from all parts of the country converging at Philadelphia, supply the fastest and most luxurious trains, and the distance from the Quaker City to the city by the sea is compassed in an hour or two. These two material questions necessarily enter into the plans when a trip of this kind is in contemplation—namely, hotel accommodations and railway facilities and comforts. For the Atlantic City meeting in June, no better facilities or comforts are offered anywhere in this country.

It is more than probable that this meeting will be more largely attended than any other in the history of the association. Several conditions conspire to give this prognostication more than a semblance of truth. The season of the year is one, the location is another, its accessibility is a third; but quite the most important of all is the unification of the profession in the state of New York, which undoubtedly will be an accomplished fact before the date fixed upon for the meeting, in so far as all the legal and business requirements necessary to accomplish the result are concerned. This latter reason certainly ought to justify the expectation that a larger number of Buffalo physicians will be present than ever before.

This meeting offers many attractions to physicians in Western New York. The journey is short, the little interruption in professional work that it will afford and the delightful entertainment in prospect, together with the reasonable rates of fare, combine to invite a large body of medical men to avail themselves of this exceptional opportunity. The picturesque Lehigh Valley Railroad offers a splendid opportunity for a view of scenery that is nowhere excelled and it should be selected, by all means, as the route of travel by those who go from this vicinity. Mr. W. B. Wheeler, Western Passenger Agent, whose address is 369 Main street, Buffalo, will gladly furnish all information necessary to the comfort and convenience of delegates, members and visitors. He can also be called up by telephone—Seneca, 2670. It is not too early for those who would secure desirable accommodations to consult Mr. Wheeler at once.

---

THE prevalence of pneumonia during the past winter has been a subject of remark throughout the Empire State. In New York City it has proved fatal in a remarkable degree especially among the aged. During the week ending March 5, the number of deaths reported to the health department from this disease was 460,—the highest recorded number of deaths from pneumonia in a single week in the history of the metropolis. The next week —namely, that ending March 12, the number of deaths had dropped to 358, a remarkable falling off, though still a high rate. The long and rigid winter has accentuated its severity with an unexampled austerity.

---

THE optometry bill was in evidence at Albany during the month of March. On the 9th, a public hearing was had before the senate committee on public health, the opposition to the bill being led by Dr. Frank Van Fleet, chairman of the legislative committee of the Medical Society of the State of New York. From Buffalo, in support of Dr. Van Fleet's contention, Dr. William C. Krauss, president of the Medical Society of the County of Erie, and Drs. Ernest Wende and Edward C. Clark, appeared before the committee. Dr. Wende is a colleague of Dr. Van Fleet on the legislative committee of the State Medical Society and is also a member of the legislative committee of the Medical Society of the County of Erie. Dr. Clark is a colleague of Dr. Wende on the latter committee. Dr. Krauss was designated to make the argument for the Buffalo delegation, which was substantially as follows:

I represent the Medical Society of the County of Erie, the third largest in the state. I am not connected with any optical house and do not fit glasses. I speak as a neurologist. The eye is intimately associated with the brain. Twelve pairs of nerves radiate from the base of the brain to the senses. Six pairs go to the eye. In the eye are motor nerves, sensory nerves, trophic nerves and nerves of special sense.

An early beginning of apoplexy is often determined by an examination of the eye. A slight headache and convergence of the eyes often means disease. To go to the eye-glass man instead of to a physician may mean delay that puts off the necessary consultation and examination until it is too late. An early recognition of a diseased state, which is first felt in the eye, often saves a life. Correcting the nerves, treating them, treating the brain or the spinal cord is the only way to accomplish much for the eyes. Defects of vision are often easily traceable to some disease that no dealer in eye-glasses can cope with by merely fitting spectacles to the eyes.

We come here today to bolster up the State Medical Law, so that only the best educated men and women shall get into the profession. We want the standard of our doctors as high as that in Europe, where they begin to teach them when they are six years of age. We must not lose sight of the fact that we should look out for the poor. All advertisements of the "greatest specialists" are not to be believed. We laugh at them as trade devices, but the poor man goes to them and often receives lasting injuries. In general, I would say that we should not make it easier to get into the medical profession. This bill is to place persons without requisite qualifications in a position legally to treat defects of the eyes, the causes of which they cannot know.

Elsewhere we print a letter from Dr. Van Fleet, the indefatigable chairman of the legislative committee of the Medical Society of the State of New York, showing that the opticians and osteopaths both failed in their endeavors to discredit the medical laws, the people and the profession—for which thanks to Dr. Van Fleet and his colleagues of the committee.

---

THE memory of Dr. William E. B. Davis, late of Birmingham, Ala., will be appropriately perpetuated by the erection of a monument to cost $2,000. The money will be furnished by the Southern Surgical and Gynecological Association of which Dr. Davis was the originator as well as founder. He not only conceived the idea, but organised the association in all its details and served as its secretary and executive officer for thirteen years. The association does well in thus testifying its appreciation of this distinguished physician, whose memory will live in spite of granite or bronze.

---

THE report of the commission to investigate the condition of the adult blind in the state of New York, of which Dr. F. Park Lewis, of Buffalo, was president, has been transmitted to the legislature and published in pamphlet form. The object in creating the commission, stated in brief, was to devise some means whereby blind persons, otherwise than those who follow professional pursuits, could be provided with such industrial occupation as would make them self-supporting.

The work of the commission was exhaustive and the report is full of interesting material relating to state provisions for the adult blind, not only in this but in other countries, and it may well attract the attention of philanthropists as well as members of the legislature. Among the recommendations made is one to establish tentatively in Buffalo, one industrial training school for blind persons over 21 years of age. Another is, that a permanent commission be appointed to direct and control the educational and industrial interests of the adult blind. It would seem advisable for the state to see to it that both suggestions are carried out.

---

A LETTER from Professor Roswell Park, published elsewhere, giving his impressions of the recent congress of German surgeons at Berlin, will be read with interest by his many thousand friends in this country. Dr. Park tells the story in his usual attractive style, which is at once simple in its English and effective in its method. It is pleasant to learn, through such a reliable observer, that our own surgeons are in nowise inferior to the German leaders, who have been held up as models, and that the American Surgical Association is not a whit behind its great German prototype. Dr. Park is expected home early in May.

---

THE Medical Society of the County of Erie, as will be observed in the proceedings published elsewhere, has ratified the action of the parent body, the Medical Society of the State of New York, relating to unification. The Erie County Medical Association took similar action at a meeting held April 18, 1904. These are the last steps required by the local organisations, and consolidation will doubtless be completed within a few weeks.

